# A Bioinformatics Approach to MicroRNA-Sequencing Analysis Based on Human Saliva Samples of Patients with Endometriosis

**DOI:** 10.3390/ijms23148045

**Published:** 2022-07-21

**Authors:** Sofiane Bendifallah, Yohann Dabi, Stéphane Suisse, Ludmila Jornea, Delphine Bouteiller, Cyril Touboul, Anne Puchar, Emile Daraï

**Affiliations:** 1 Department of Obstetrics and Reproductive Medicine, Hospital Tenon, Sorbonne University, 4 rue de la Chine, 75020 Paris, France; yohann.dabi@gmail.com (Y.D.); cyril.touboul@gmail.com (C.T.); anne.puchar@aphp.fr (A.P.); emile.darai@aphp.fr (E.D.); 2 Clinical Research Group (GRC) Paris 6: Endometriosis Expert Center (C3E), Sorbonne University (GRC6 C3E SU), 75020 Paris, France; 3 Cancer Biology and Therapeutics, Centre de Recherche Saint-Antoine (CRSA), Sorbonne University, INSERM UMR_S_938, 75020 Paris, France; 4 Ziwig Health, 19 rue Reboud, 69003 Lyon, France; stephane@ziwig.com; 5 Paris Brain Institute-Institut du Cerveau-ICM, Sorbonne University, Inserm U1127, CNRS UMR 7225, AP-HP-Hôpital Pitié-Salpêtrière, 75013 Paris, France; ludmila.jornea@icm-institute.org; 6 Gentoyping and Sequencing Core Facility, iGenSeq, Institut du Cerveau et de la Moelle Épinière, ICM, Hôpital Pitié-Salpêtrière, 47-83 Boulevard de l’Hôpital, 75013 Paris, France; delphine.bouteiller@icm-institute.org

**Keywords:** endometriosis, miRNA, NGS, bioinformatics, saliva

## Abstract

Endometriosis, defined by the presence of endometrium-like tissue outside the uterus, affects 2–10% of the female population, i.e., around 190 million women, worldwide. The aim of the prospective ENDO-miRNA study was to develop a bioinformatics approach for microRNA-sequencing analysis of 200 saliva samples for miRNAome expression and to test its diagnostic accuracy for endometriosis. Among the 200 patients, 76.5% (n = 153) had confirmed endometriosis and 23.5% (n = 47) had no endometriosis (controls). Small RNA-seq of 200 saliva samples yielded ~4642 M raw sequencing reads (from ~13.7 M to ~39.3 M reads/sample). The number of expressed miRNAs ranged from 1250 (outlier) to 2561 per sample. Some 2561 miRNAs were found to be differentially expressed in the saliva samples of patients with endometriosis compared with the control patients. Among these, 1.17% (n = 30) were up- or downregulated. Among these, the F1-score, sensitivity, specificity, and AUC ranged from 11–86.8%, 5.8–97.4%, 10.6–100%, and 39.3–69.2%, respectively. Here, we report a bioinformatic approach to saliva miRNA sequencing and analysis. We underline the advantages of using saliva over blood in terms of ease of collection, reproducibility, stability, safety, non-invasiveness. This report describes the whole saliva transcriptome to make miRNA quantification a validated, standardized, and reliable technique for routine use. The methodology could be applied to build a saliva signature of endometriosis.

## 1. Introduction

MicroRNAs (miRNAs) are small, highly conserved non-coding RNAs with a length of about 22 nucleotides which bind to the 3′-untranslated region (3′-UTR) of target messenger RNAs (mRNAs), thus regulating gene expression post-transcriptionally through RNA degradation and/or translational inhibition [1,2]. Schematically, miRNA biosynthesis involves several steps: (i) they are first transcribed from genes in intronic regions of coding or non-coding transcripts, or coded from exons under the action of the RNA polymerase II, generating hundreds of duplex nucleotide-long primary miRNAs (pri-miRNA); (ii) the pri-miRNA is subsequently cleaved by a complex formed by an RNase III enzyme, Drosha, RNA binding cofactor and Pasha to generate precursor miRNA (pre-miRNA); and (iii) then, the pre-miRNAs are transported from the nucleus to the cytoplasm using exportin 5 where the duplex is cleaved by Dicer and helicase to form mature miRNAs [2,3]. The miRNAs are subsequently incorporated into an RNA silencing complex (RISC) that regulates post-translational modifications through binding to the 3′ untranslated region (3′ UTR) of the target messenger-RNA (mRNA). Finally, the miRNAs are released from the cells into circulation using various carriers such as Argonaute, nucleophosmin 1, high-density lipoproteins or extracellular vesicles (exosomes) with a distribution in human fluids where they can be detected [4,5].

Numerous studies have demonstrated the relevance of evaluating miRNA expression in cancers and benign pathologies to provide insights into the molecular mechanisms of disease onset and progression [3,6,7,8,9,10]. For these reasons, sequencing has been applied to biomarker discovery for a variety of diseases, such as endometriosis, but with some limitations especially for saliva samples. Indeed, several sources of errors can be introduced during a sequencing study such as (i) an underpowered cohort [3,11,12], (ii) sample extraction, (iii) library preparation (12, 15–18) [7,13,14,15], and (iv) sequencing technique. Overall, these errors can lead to over- or underestimation of the molecule expression or a one-size-fits-all approach with inadequate analysis [8,9,12,14,15,16].

Therefore, the goal of the prospective ENDO-miRNA study was to develop a bioinformatics approach for microRNA-sequencing analysis of 200 saliva samples for miRNAome expression and to test its diagnostic accuracy for endometriosis.

## 2. Material and Methods

### 2.1. Study Population

We used data from the prospective “ENDOmiARN” study (ClinicalTrials.gov Identifier: NCT04728152). Data collection and analysis were carried out under Research Protocol n° ID RCB: 2020-A03297-32. We obtained signed informed consent from all participants in the study. The experimental protocol was approved by Ethics committee le comité de protection des personnes (C.P.P.) Sud-Ouest et Outre-Mer 1 (CPP 1-20-095 ID 10476).

The ENDOmiARN study included 200 saliva samples obtained from patients with chronic pelvic pain suggestive of endometriosis. All the samples were collected between January 2021 and June 2021. Analysis was performed blinded to the surgical and imaging findings. The patients with endometriosis were stratified according to the revised American Society of Reproductive Medicine (rASRM) classification [17]. The main characteristics of the patients included in the ENDOmiARN study are displayed in Table 1.

### 2.2. Saliva Sample Collection

The saliva samples (2 mL) were collected in an all-in-one system including a nucleic acid stabilizing solution for collection, stabilization and transportation (OME 505, DNA Genotek Inc., 2 Beaverbrook Road Ottawa, ON, Canada K2K 1L1) using an at-home kit (https://www.dnagenotek.com/row/products/collection-microbiome/omnigene-oral/OME-505.html, accessed on 1 January 2021). Subjects were asked to refrain from eating, drinking, smoking, or chewing gum for 30 min before the saliva sample was taken. All the samples were stored at room temperature prior to shipping.

### 2.3. RNA Sample Extraction, Preparation and Quality Control

RNA was isolated from each saliva sample using the miRNeasy Kit (Qiagen, Inc., Germantown, MD, USA) according to the manufacturer’s instructions [6,8,9]. In accordance with DNA Genotek process of extraction, a systematic centrifugation was performed at 13,300× *g* for 3 min. RNA quality was assessed using the Agilent Technologies TapeStation 2200. RNA-sequencing libraries were prepared using the QIAseq miRNA Library Kit (Qiagen, Hilden, Germany) according to the manufacturer’s instructions. Samples were indexed in batches of 96, with a targeted sequencing depth of 17 million reads per sample. Sequencing was performed using 100 base single-end reads, using an Novaseq6000 sequencer (Illumina, San Diego, CA, USA) [14,18]. The process used is the one summarized in the previously published work by Potla et al. [13].

## 3. Bioinformatics

### 3.1. Raw Data Preprocessing (Raw, Filtered, Aligned Reads) and Quality Control

Sequencing reads were processed using the data processing pipeline. FastQ files were trimmed to remove adapter sequences using Cutadapt version v.1.18 and were aligned using Bowtie version 1.1.1 to the following transcriptome databases: the human reference genome available from NCBI (https://www.ncbi.nlm.nih.gov/genome/guide/human/, accessed on 1 January 2021), and miRBase21 (miRNAs) using the MirDeep2 v0.1.0 package. The raw sequencing data quality was assessed using FastQC software v0.11.7 [14,15,19,20,21].

### 3.2. Differential Expression Analysis of miRNA

miRNA expression was quantified by miRDeep2 v0.1.0 [22]. Differential expression tests were then conducted in DESeq2 for miRNAs with read counts in ≥1 of the samples. DESeq2 V1.20 integrates methodological advances with several novel features to facilitate a more quantitative analysis of comparative RNA-seq data using shrinkage estimators for dispersion and fold change [23]. The resulting matrix was filtered for expressed miRNAs and normalized using Z-score normalization [24]. miRNAs were considered as differentially expressed if the absolute value of log2-fold change was >1.5 (upregulated) and <0.5 (downregulated). The *p* value adjusted for multiple testing was <0.05 [23].

### 3.3. miRNA Diagnostic Accuracy

To evaluate the diagnostic accuracy of each miRNA biomarker, sensitivity, specificity, an ROC analysis was performed, and the ROC AUC was calculated [25,26].

Additional statistical analysis was based on the Chi^2^ test as appropriate for categorical variables. Values of *p* < 0.05 were considered to denote significant differences. Data were managed with an Excel database (Microsoft, Redmond, WA, USA) and analyzed using R 2.15 software, available online (http://cran.r-project.org/, accessed on 1 January 2021).

## 4. Results

### 4.1. Description of the ENDOmiARN Cohort

Among the 200 patients, 76.5% (n = 153) had confirmed endometriosis and 23.5% (n = 47) had no endometriosis (controls). In the endometriosis group, 52% (80) of the patients had rASRM stages I–II and 48% (73) had stage III–IV. The control group consisted of various benign pathologies with 51% (24) of the women having no abnormality. These were defined as “discordant” (or complex) patients corresponding to women with symptoms suggestive of endometriosis without clinical or MRI features of endometriosis and no endometriosis lesions discovered during laparoscopic inspection (Table 1).

### 4.2. Global Overview of the Saliva miRNAome

**Figure 1:**
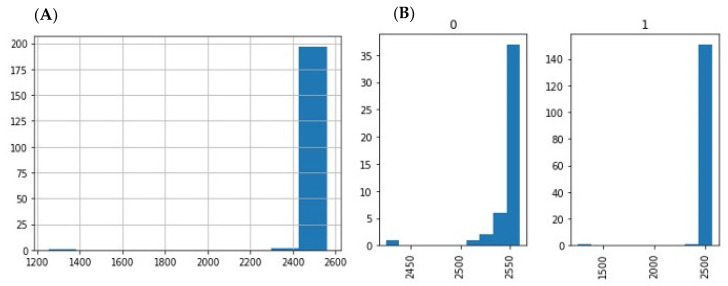
(**A**) Distribution of expressed miRs in the 200 saliva samples; (**B**) distribution of expressed miRNAs in the samples by diagnosis.

**Figure 2:**
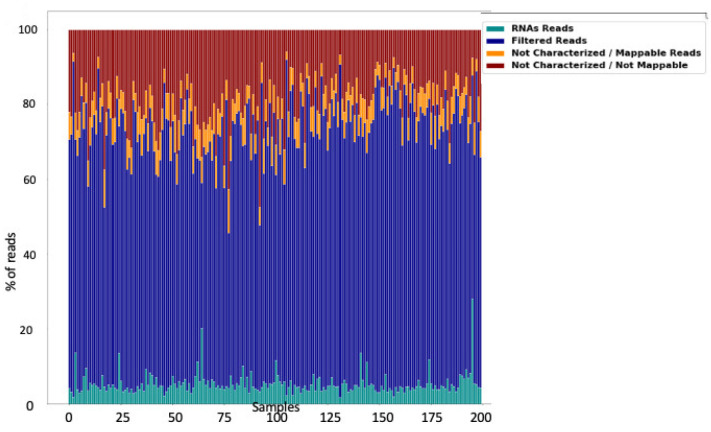
Overall composition of processed reads for saliva sample RNA reads = miRNAs + piRNAs + rRNAs + tRNAs + mRNAs + other; filtered reads = reads with no adapters + reads with low quality bases + reads too short; not characterized/mappable reads = mapped reads to GRCh38 that could not be characterized as a particular type; not characterized/not mappable reads = reads that could not be mapped.

Small RNA-seq of 200 saliva samples yielded ~4 642 M raw sequencing reads (from ~13.7 M to ~39.3 M reads/sample). Pre-filtering and filtering steps retained 70% (~3205 M) of initial raw reads. The majority of filtered reads were of short read length. Quantification of filtered reads and identification of known miRNAs yielded ~190 M sequences to be mapped to 2561 known miRNAs from miRBase v21. The number of expressed miRNAs ranged from 1250 (outlier) to 2561 per sample. The distribution of expressed miRNAs in the 200 saliva samples and the overall composition of processed reads are shown in Figure 1A,B and Figure 2.

### 4.3. miRNA Expression in Patients with and without Endometriosis

**Figure 3:**
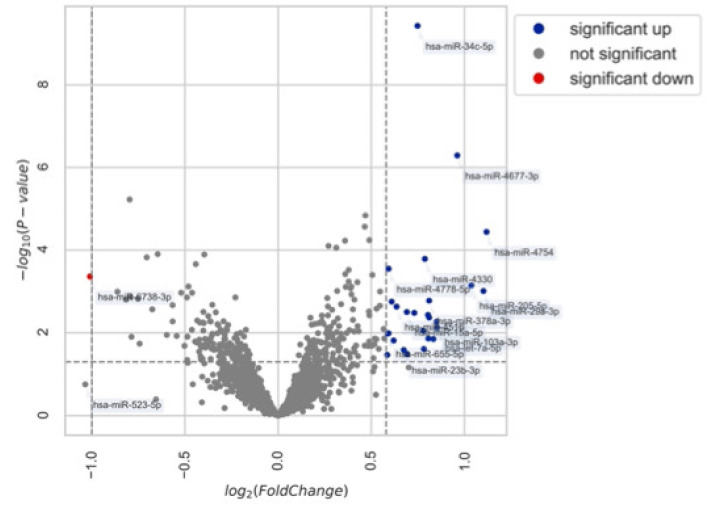
Volcano plot of expressed miRNAs in saliva for endometriosis.

**Figure 4:**
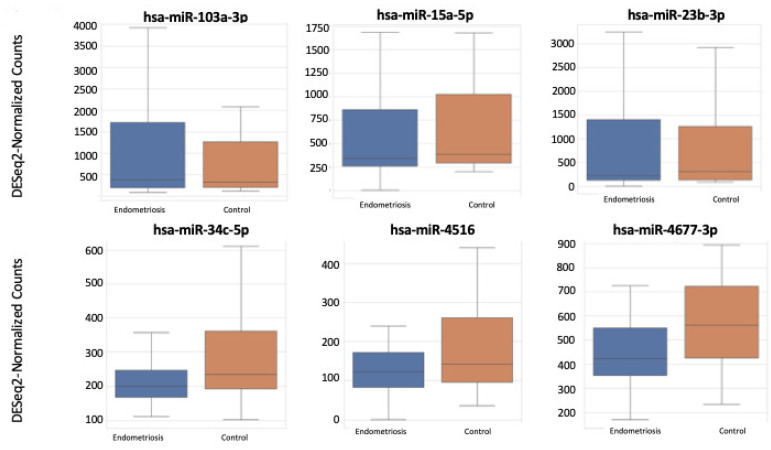
Small RNA-seq defines differentially expressed miRNAs in the saliva of endometriosis patients.

Of the miRNAs, 2561 were found to be differentially expressed in the saliva samples of patients with endometriosis, compared with the control patients. Among these, 1.17% (n = 30) were up- or downregulated. Figure 3 shows a volcano plot of the miRNAs expressed in endometriosis. Among the 30 regulated miRNAs, only three (hsa-miR-34c-5p, hsa-miR-4677-3p, hsa-miR-655-5p) had an AUC > 0.6. The top 10 differentially expressed miRNA patterns in the endometriosis and control are reported in Figure 4.

### 4.4. Diagnostic Accuracy of Regulated miRNAs

The diagnostic metrics for endometriosis in the differentially expressed miRNAs in the saliva samples (n = 30) are reported in Table 2. Among these, the F1-score, sensitivity, specificity, and AUC ranged from 11–86.8%, 5.8–97.4%, 10.6–100%, and 39.3–69.2%, respectively.

For AUC criteria, 90% (n = 27), and 10% (n = 3) had a value ranging between 36.3–59% and ≥60%, respectively.

For the F1-score, 80% (n = 24) and 20% (n = 6) had a value ranging between 0–79%, and ≥80%, respectively

For sensitivity, 80% (n = 24) and 20% (n = 6) had a value ranging between 0–79%, and ≥80%, respectively

**Figure 5:**
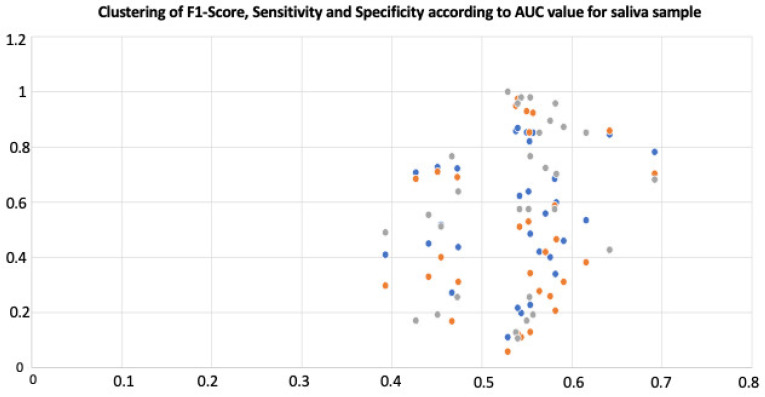
Clustering of accuracy values. In blue: F1-Score; In orange: Sensitivity; In grey: Specificity.

For specificity, 70% (n = 21) and 10% (n = 9) had a value ranging between 0–79%, and ≥80%, respectively. The clustering of the accuracy values is reported in Figure 5.

## 5. Discussion

To the best of our knowledge, this is the first report detailing the miRNAome of 200 saliva samples from patients with and without endometriosis included in a prospective study: the ENDOmiARN study [3,11,16,27]. In addition, we report a bioinformatics approach to saliva miRNA sequencing and analysis and underline the advantages of using saliva over blood in terms of ease of collection, reproducibility, stability, safety, non-invasiveness, and cost-effectiveness [6,8,10,22,28,29,30,31].

Preliminary results about the use of saliva RNAs as diagnostic biomarkers have previously been reported, mainly for cancer [6,8,32], systemic disease, and forensic casework [6,9,28,33]. However, the quality of the methodology and yield issues of these studies overall are debatable [34]. In a recent literature review of miRNAs for the non-invasive diagnosis of endometriosis, Monnaka et al. underlined that none of the 449 reports investigated miRNAs in saliva [11]. Therefore, since (i) no scientifically proven salivary biomarkers for endometriosis have been reported, and (ii) the applicability of such biomarkers has been poorly explored, the concept of extracting and identifying miRNAs from saliva samples for the reliable identification of endometriosis is challenging [35,36]. The main obstacle to using miRNAs is their stability and susceptibility to degradation. This has always been an issue for mRNA-based gene expression analysis and a potential source of bias for reproducibility [28,34,37]. This point was highlighted, for example, for forensic routine applications using miRNA, because biological stains from forensic casework are often altered by ambient moisture and temperature, UV light, suboptimal environmental pH, which all have the potential to degrade the miRNA beyond usability [28]. In this setting, Patel et al. demonstrated that Oragene•RNA solution could preserve and stabilize RNA collected from saliva to produce high yields of good quality RNA for subsequent downstream applications and/or analyses [34]. The authors reported that the RNA yield remained fairly constant between matched samples from each donor when stored for 48 h at room temperature [34]. In addition, they explored the differences in the total RNA yield from donors over a 3-day period, but also sought to examine the potential differences in expression between commonly used mRNA and miRNA endogenous controls [34]. Although the total RNA from each donor varied over the days, probably due to bacterial RNA, the abundance of the mammalian RNA normalizers (snU6 small RNA, 18S rRNA, GAPDH mRNA and let-7b miRNA) remained stable [34]. In the current study, 200 saliva samples were collected according to the manufacturer’s guidelines (Oragene) and stored at room temperature prior to shipping and analysis. Interestingly, we found that the quantification of filtered reads and identification of miRNAs yielded ~190 M sequences to be mapped to 2561 known miRNAs. The total reads ranged from 13 to 39 million with a mean of 23 million. Among these, the miRNA reads ranged from 272,322 to 6 million with a mean of 949,893 (Figure 2A,B). These results are in concordance with previous reports demonstrating that the salivary transcriptome is abundant and stable, consisting of thousands of mRNAs and miRNAs [6,9,10,28,34,38]. In this setting, Courts et al. also confirmed that miRNAs are especially relevant because they are stable and easy to collect and analyze, and validated their use in standard forensic medicine [28]. Using the Oragene•RNA kit, we demonstrated (i) the stability and consistency of miRNA reads for the 200 samples whatever the conditions of sampling and transport, (ii) the reproducibility and efficiency of such techniques since all the 200 samples were usable for sequencing, and (iii) a routine bioinformatics approach. In the present study, diagnostic accuracies according to the F1-score, sensitivity, specificity and AUC ranged from 11–86.8%, 5.8–97.4%, 10.6–100%, and 39.3–69.2%, respectively. In addition, we identified 30 miRNAs up- and downregulated with a high heterogeneity in terms of accuracy.

Although the use of saliva for miRNA identification could be a potential non-invasive solution to overcome current barriers to the diagnosis of endometriosis, the critical step is the transition from expression data to candidate selection, which is always somewhat arbitrary. In this setting, salivary miRNAs have been reported to be of great interest as diagnostic biomarkers especially in cancer [6,10,32]. However, as there are no fixed rules about which criteria to apply to select a miRNA candidate, we developed a bioinformatics approach for miRNA accuracy: among the 2561 miRNAs identified, 30 were up- or downregulated, underpinning the use of new mathematical methods and artificial intelligence to overcome the limits of classic logistic regression. Indeed, in agreement with Lopez-Rincon et al., it is illusory to imagine that a few mi-RNAs could reflect the heterogeneity of a multifactorial disorder such as endometriosis, characterized by various phenotypes and for which the various pathways implicated in its genesis are poorly understood [7,15,39]. We thus used a new statistical tool, machine learning, to overcome the accuracy limitations and design a potential diagnostic signature [7,9,10,15,30].

In the present study, we analyzed 200 plasma samples for miRNA expression and diagnostic accuracy. However, there are several unsolved issues that might hinder the broad acceptance of a miRNA-based signature. The miRNAome, perhaps even more than the transcriptome, is highly context dependent, and it is conceivable that certain non- physiologic or pathologic conditions might alter the expression levels of miRNAs for body-fluid identification. It will therefore be necessary to test whether the expression of candidate miRNAs for body-fluid identification are influenced by biologic processes or conditions such as the menstrual cycle phase or previous hormonal treatment [12,40]. In this setting, Vanhie et al. and Moustafa al. reported no impact on miRNA expression according either to hormonal treatment or menstrual cycle phases in contrast to data obtained from endometrial biopsies [12,40]. This apparent discrepancy could be linked to the modalities of miRNA release into bodily fluids that could vary depending on the organ and the tumor. In the ENDOmiARN study, two different body fluids were assessed: serum and saliva. This choice was mostly driven by the need for stability in the miRNAs detected to provide a reliable diagnostic tool. Indeed, while several studies have observed differences in miRNA expression in tissues according to the menstrual phase, mainly at endometrial level [41,42], no such cyclic differences were observed in the plasma of healthy women [43]. One hypothesis is that changes in miRNA expression at the endometrium level regulate gene expression locally but are insufficient to cause detectable systemic changes [3]. The other reason to opt for saliva was its easy availability, including in a home self-sampling setting and including virgin patients not examined during gynecological appointments. Another issue is the variations of miRNA expression analysis according to the next-generation sequencing (NGS) technique used. Indeed, several different methods and devices for miRNA extraction, reverse transcription and quantification from NGS to microarray analysis have been advocated leading to differences in results [13,31,34]. However, as underlined by Agrawal et al., we believe that the standardized NGS procedure we describe here is optimal for endometriosis since it is currently the gold standard approach for profiling nucleic acid, including miRNAs [3]. In addition, miRNAs are just one of several classes of small, ncRNAs with regulatory functions, and there is no reason to exclude these RNAs from endometriosis analyses. Therefore, miRNA analysis may represent an interim strategy until more is known about other small RNAs, and once a comprehensive small-RNA analysis is available, it is likely to replace miRNA only analysis [44]. Eventually, our results require external validation supporting temporal and geographic validation of for mi RNA quantification and sequencing reproducibility; that is the goal of an ongoing study [45].

## 6. Conclusions

Endometriosis affects about 190 million women worldwide, representing a healthcare burden equivalent to diabetes [46]. Endometriosis is a representative example of a multifactorial and not completely understood, chronic disease. To understand the various signaling pathways involved in this complex disease, analysis of the entire miRNome currently available is mandatory. This report describes the whole saliva transcriptome to make miRNA quantification a validated, standardized, and reliable technique for routine use. The methodology could be applied to build a saliva signature of endometriosis and to solve other issues of this debilitating disorder—various clinical phenotypes, infertility-associated endometriosis—as well as to evaluate the potential theragnostic value of miRNA expression. Finally, beyond endometriosis, our methodology can be applied to other chronic diseases with the goal of developing a noninvasive, quick and reliable tool to improve diagnosis, management and to select patients according to therapeutic medical and/or surgical response.

## Figures and Tables

**Table 1:** Demographic characteristics of the patients included in the ENDOmiARN cohort. BMI: body mass index; rASRM: revised American Society for Reproductive Medicine.

	Controls N = 47	Endometriosis N = 153	
Age years (mean ± SD)	30.92 ± 13.79	31.17 ± 10.78	0.19
BMI (body mass index) (mean ± SD)	24.84 ± 11.10	24.36 ± 8.38	0.52
rASRM classification			-
- I–II	-	80 (52%)	
- III–IV	-	73 (48%)	
Control diagnoses			
- No abnormality	24 (51%)	-	-
- Leiomyoma	1 (2%)		
- Cystadenoma	5 (11%)		
- Teratoma	11 (23%)		
- Other gynecological disorders	6 (13%)		
Dysmenorrhea	100%	100%	
Abdominal pain outside menstruation			
- Yes	21 (66%)	89 (71%)	0.69
Patients with pain suggesting sciatica	10 (31%)	70 (56%)	0.02
Dyspareunia intensity at VAS (mean ± SD)	4.95 ± 3.52	5.28 ± 3.95	<0.001
Patients with lower back pain outside menstruation	20 (62%)	101 (81%)	0.049
Intensity of pain during defecation at VAS (mean ± SD)	2.84 ± 2.76	4.35 ± 3.47	<0.001
Patient with right shoulder pain during menstruation	3 (9%)	26 (21%)	0.21
Intensity of urinary pain during menstruation at VAS (mean ± SD)	2.84 ± 2.76	4.35 ± 3.36	<0.001
Patient with blood in the stools during menstruation	4 (12%)	30 (24%)	0.24
Patient with blood in urine during menstruation	8 (25%)	21 (17%)	0.41

**Table 2:** Diagnostic metrics for endometriosis for differentially expressed miRNAs in the saliva samples (n = 30).

miRNA	Regulation	AUC	F1-Score	Sensitivity	Specificity
hsa-let-7a-5p	UP	0.473	0.721	0.69	0.255
hsa-let-7i-5p	UP	0.451	0.726	0.71	0.191
hsa-miR-101-3p	UP	0.554	0.227	0.129	0.979
hsa-miR-103a-3p	UP	0.582	0.339	0.206	0.957
hsa-miR-142-3p	UP	0.529	0.11	0.058	1
hsa-miR-146a-5p	UP	0.538	0.857	0.948	0.128
hsa-miR-15a-5p	UP	0.571	0.558	0.419	0.723
hsa-miR-16-5p	UP	0.427	0.707	0.684	0.17
hsa-miR-199a-3p	UP	0.467	0.271	0.168	0.766
hsa-miR-203b-5p	UP	0.474	0.436	0.31	0.638
hsa-miR-205-5p	UP	0.544	0.197	0.11	0.979
hsa-miR-223-3p	UP	0.455	0.517	0.4	0.511
hsa-miR-23a-3p	UP	0.54	0.216	0.123	0.957
hsa-miR-23b-3p	UP	0.564	0.42	0.277	0.851
hsa-miR-24-3p	UP	0.542	0.622	0.51	0.574
hsa-miR-26a-5p	UP	0.54	0.868	0.974	0.106
hsa-miR-29a-3p	UP	0.552	0.638	0.529	0.574
hsa-miR-29c-3p	UP	0.441	0.449	0.329	0.553
hsa-miR-3191-3p	UP	0.55	0.852	0.929	0.17
hsa-miR-34c-5p	UP	0.642	0.844	0.858	0.426
hsa-miR-378a-3p	UP	0.554	0.484	0.342	0.766
hsa-miR-4330	UP	0.393	0.409	0.297	0.489
hsa-miR-4516	UP	0.581	0.684	0.587	0.574
hsa-miR-4677-3p	UP	0.692	0.781	0.703	0.681
hsa-miR-4754	UP	0.553	0.82	0.852	0.255
hsa-miR-4778-5p	UP	0.583	0.598	0.465	0.702
hsa-miR-523-5p	DOWN	0.576	0.4	0.258	0.894
hsa-miR-655-5p	UP	0.616	0.534	0.381	0.851
hsa-miR-6726-5p	UP	0.557	0.851	0.923	0.191
hsa-miR-6738-3p	DOWN	0.591	0.459	0.31	0.872

## Data Availability

The authors state that the data used are from the prospective ENDOmiARN study (ClinicalTrials.gov Identifier: NCT04728152). Data collection and analysis were carried out under Research Protocol n° ID RCB: 2020-A03297-32.
